# Transient emotional events and individual affective traits affect emotion recognition in a perceptual decision-making task

**DOI:** 10.1371/journal.pone.0171375

**Published:** 2017-02-02

**Authors:** Emilie Qiao-Tasserit, Maria Garcia Quesada, Lia Antico, Daphne Bavelier, Patrik Vuilleumier, Swann Pichon

**Affiliations:** 1 Laboratory for Behavioral Neurology and Imaging of Cognition, Department of Neuroscience, Medical School, University of Geneva, Geneva, Switzerland; 2 Swiss Center for Affective Sciences, University of Geneva, Geneva, Switzerland; 3 Faculty of Psychology and Educational Sciences, University of Geneva, Geneva, Switzerland; 4 Department of Brain & Cognitive Sciences, University of Rochester, Rochester, New York, United States of America; University of Bologna, ITALY

## Abstract

Both affective states and personality traits shape how we perceive the social world and interpret emotions. The literature on affective priming has mostly focused on brief influences of emotional stimuli and emotional states on perceptual and cognitive processes. Yet this approach does not fully capture more dynamic processes at the root of emotional states, with such states lingering beyond the duration of the inducing external stimuli. Our goal was to put in perspective three different types of affective states (induced affective states, more sustained mood states and affective traits such as depression and anxiety) and investigate how they may interact and influence emotion perception. Here, we hypothesized that absorption into positive and negative emotional episodes generate sustained affective states that outlast the episode period and bias the interpretation of facial expressions in a perceptual decision-making task. We also investigated how such effects are influenced by more sustained mood states and by individual affect traits (depression and anxiety) and whether they interact. Transient emotional states were induced using movie-clips, after which participants performed a forced-choice emotion classification task with morphed facial expressions ranging from fear to happiness. Using a psychometric approach, we show that negative (vs. neutral) clips increased participants’ propensity to classify ambiguous faces as fearful during several minutes. In contrast, positive movies biased classification toward happiness only for those clips perceived as most absorbing. Negative mood, anxiety and depression had a stronger effect than transient states and increased the propensity to classify ambiguous faces as fearful. These results provide the first evidence that absorption and different temporal dimensions of emotions have a significant effect on how we perceive facial expressions.

## Introduction

Emotions and mood provide powerful prisms which shape the way individuals interpret social information and their environment. Depending on their affective state, people may see the glass as either half full or half empty when evaluating the same situation. But although these statements seem well known and almost trivial, little research has been conducted to characterize the impact and temporal aspects of such phenomena and to systematically compare the influence of negative and positive affective states on perception. Moreover, most priming studies assume that these biases are short-lived and do not persist more than a fraction of second. Here we addressed this issue by using a simple forced-choice categorization task where subjects evaluated facial expressions morphed between fear and happiness after being exposed for several minutes to negative, positive, or neutral movie clips. Our aim was to measure bias in expression categorization induced by transient affective states and estimate whether such bias persists during the entire duration of the categorization task. We also tested whether such biases depend on more persistent affective states related to individual personality characteristics such as positive and negative mood as well as sub-clinical levels of anxiety and depression.

Unlike persistent affective states such as mood, emotions are generally conceived as short-lived events of physiological, motivational, and psychological changes that have a definite cause, a distinctive cognitive and experiential content, and a rapid decay after elicitation [1,2]. Some studies using EEG have characterized the temporal unfolding of brain responses to emotional events (pictures) and pointed to differential dynamics at the timescale of the second for different emotions [3,4]. However, little is known about changes in behavior and brain activity occurring over several seconds or minutes after emotional events [5]. Likewise, the literature on affective priming has mostly focused on very brief influences of emotional stimuli on perceptual and cognitive processes by employing cue-target paradigms similar to the ones used in the spatial or psycholinguistic domains. In this framework, affective priming typically designates the effect produced by a brief emotional stimulus (the prime) on the evaluation of subsequent information (the target) [6,7]. Affective priming has also been investigated using subliminal masked priming [8,9]. Such affective priming effects are inherently short-lived and dissipate after a fraction of a second [10–12].

However, little has been documented on situations where emotional information is presented for longer periods of time, such as when subjects watch a movie, listen to music, or imagine an emotional event before evaluating a target. Such situations may ‘infuse’ affective states and impact cognitive states over longer periods of time, as proposed in the affect infusion model [13]. The affect infusion model has specifically considered that such extended affective states can modulate the encoding, retrieval, and selective use of information. Such extended states can influence the interpretation of subsequent events even after transient emotions have ‘faded away’ and their experiential content has vanished [14–16]. For instance, watching a movie depicting either a funeral or a child playing, will imbue a subsequent neutral scene with either negative or positive meaning [17,18]. This phenomenon, referred to as the Kuleshov effect used in film editing, can also operate on the perception of neutral faces, and it implies some transfer of emotional or mental content between one movie scene and the perceived facial expression of a character in the next scene [17]. However, few empirical studies have directly investigated the influence of affective states on face perception [5,17–19]. To our knowledge, no study has investigated whether a transient emotion induction procedure can induce interpretation biases for facial expressions, whether the induced biases quickly vanish after induction or persist over several minutes, and whether such interpretation biases vary according to the valence of emotional state. These questions constituted the first objective of the current study.

In a related but different literature, persistent negative emotional states associated with low mood, depression, or anxiety, have frequently been shown to yield a negative interpretation bias. These traits tend to favor negative over positive interpretations [20–22] of ambiguous positive-negative faces [23–26] or ambiguous semantic information [27]. A negative interpretation bias is a type of cognitive bias where there is a tendency to interpret ambiguous stimuli in a negative manner when others would favor positive interpretations [20,21]. According to cognitive theories of depression and anxiety, negative cognitive biases have a major, perhaps causal role in the development and maintenance of depressive and anxious states [28].

Therefore, the second objective of our study was to measure subject-wise interpretation biases for facial expressions induced by emotional states in relation to individual differences in mood, anxiety, and depression indices. Indeed, persistent negative states, such as depression, are associated with anhedonia and various emotional biases in perception and attention [29,30]. Given that emotional blunting is frequent in depressed patients, whereas heighted emotional reactivity is commonly associated with anxiety [31], we expected a decreased or exaggerated emotion induction effect in subjects with higher depression or higher anxiety scores, respectively.

Finally, another important factor likely to influence the impact of emotional signals on cognition and perception is the degree of absorption while individuals process emotional information. Yet, to our knowledge, the effect of absorption has been little characterized experimentally. With respect to affects, absorption refers to the extent to which individuals allow themselves to be drawn into an emotional experience. Tellegen and Atkinson (1974) defined absorption as a state of “total attention, involving a full commitment of available perceptual, motoric, imaginative and ideational resources” [32]. Recent evidence indicates that trait absorption is related to enhanced emotional picture processing [33]. Thus, a high absorption state during the processing of emotional information may potentiate more vigorously subsequent interpretation biases than a low absorption state.

Accordingly, several recent studies using neuroimaging techniques have shown that exposure to emotional stimuli using movies [5,34,35] or other emotion-inducing procedures [36] can produce lasting changes in patterns of brain activity, both during resting state [34] and during unrelated perceptual or semantic tasks [19,35,36]. In one study using faces [5], amygdala response to fearful facial expressions (compared to neutral) was enhanced for several minutes following exposure to fear-inducing movies, but attenuated following joyful movies. Furthermore, this study found that such effects were stronger for highly absorbing emotional movies [5]. It remains unknown, however, whether these neural changes are mirrored by measurable behavioural changes in facial expression recognition.

Hence, the goal of the present work was to investigate how transient positive and negative emotions induced by movie-clips influence emotion discrimination for negative and positive facial expressions, using morphed stimuli ranging from pure fear to pure happiness. We also tested how individual characteristics related to persistent mood, personality traits, and experiential absorption would modulate such influences. We predicted that negative and positive emotional states, both transient and persistent, should result in state-congruent interpretation biases for ambiguous facial expressions along the negative-positive continuum.

## Methods

### Subjects

45 healthy right-handed volunteers participated in this study (22 females, mean age±std 24.1±3.5, range = 18–32). One participant gave random responses was excluded for failing to comply with the instructions. All participants gave their informed written consent before participating in the study, which was carried out in accordance with The Code of Ethics of the World Medical Association (Declaration of Helsinki) for experiments involving humans. The study protocol, inclusion criteria, and consent procedure were reviewed and approved by the ethical committee of the University of Geneva. Participants were compensated for their participation in this study. None had a history of psychiatric or neurological disease, and none took drugs or medication at the time of testing.

### Procedure

**Fig 1:**
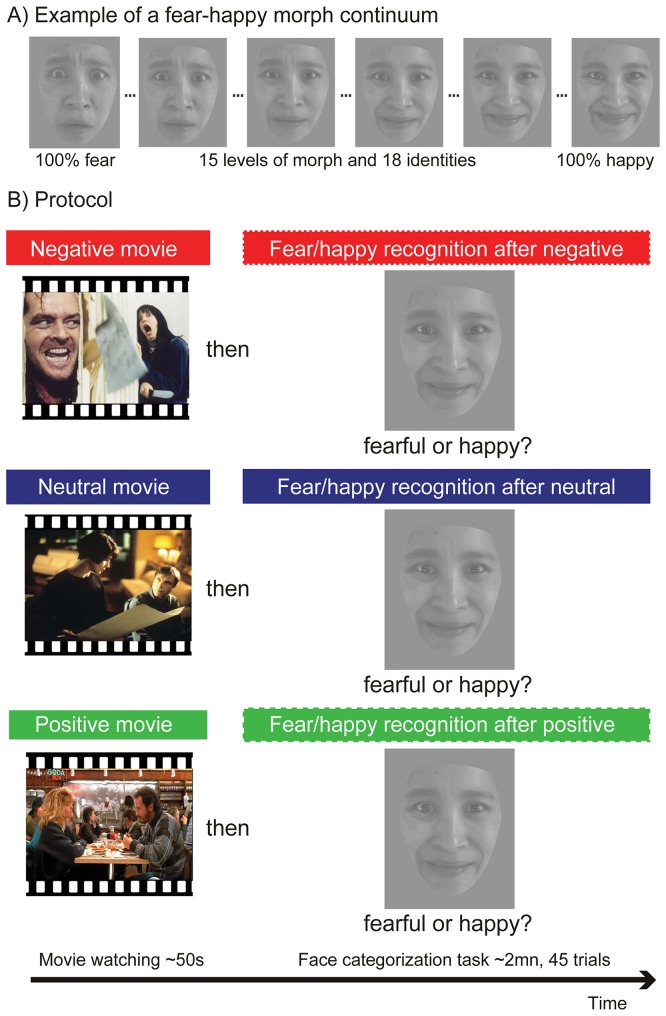
Protocol. A) Example of a fear-happy morph continuum in facial expression, ranging from pure fear to pure happiness. B) During the task, participants watched negative, neutral or positive movie clips, and subsequently performed a decision-making task where they categorized morphed faces as fearful or happy. Note that these face images are examples similar but not identical to the faces presented in the actual tasks which used KDEF faces, and are therefore for illustrative purposes only. We have received informed consent according to Plos guidelines from the individual portrayed here.

During the experiment, participants performed two intermixed tasks arranged in 18 short sessions, all given in a single day and separated by brief rest breaks. At the beginning of each session, subjects watched a positive, negative, or neutral movie clip (~ 50sec–1 min) that was immediately followed by a decision-making task on a series of 15 faces (~2mn). Each face was presented during 500ms and preceded by a fixation cross (500ms). They had to indicate if the face expressed fear or happiness (2 alternatives forced-choice task, Fig 1) without imposed response time limit. Facial expressions were morphed from fear to happiness (see below). To keep the goal of the experiment implicit, participants were told that they would participate in two different studies performed in alternating sessions, to make the experiment less repetitive. The objective of the first study would be to measure their emotional reaction to short negative/threatening, positive/joyful, or neutral film excerpts via their skin conductance response. Participants were instructed to get immersed in the movies as much as possible without being critical. They were told that the objective of the second study would be to measure their ability to recognize fear and happiness in ambiguous morphed faces.

### Movie stimuli

We used 6 movie clips per emotion conditions (negative, positive, and neutral). Movies were selected from a standard database of emotional movies [37]. Each session comprised one movie excerpt followed by 45 facial morphs. Previous studies using the same movies confirmed reliable difference in their valence [34], and showed sustained changes in brain activity and peripheral autonomic state (skin conductance and pupil size) induced by both negative and positive emotional movies (relative to neutral ones), extending for several minutes after the movies [5]. Examples of movies included *The Shining* (negative), *When Harry met Sally* (positive), or documentaries such as *Cosmos Mysteries* (neutral); see full list in [34]. We equated luminance across movies. Mood's median tests confirmed that audiovisual features were matched across contexts [luminance (p = .13), motion (p = .69), spatial frequency (p = 1), acoustic intensity and power (p>.51)]. To potentiate mood induction, movies from the same valence were grouped in successive sessions intermixed with the decision-making task, but the order of movies within emotional contexts was randomized between subjects. Half of the participants saw negative movies first, followed by neutral and positive movies. Another half of participants performed the task in the reverse order. Neutral movies were kept in the middle to facilitate the transition between negative and positive states.

### Face stimuli

We generated happy and fearful composite images from 18 faces (9 females) showing a fearful facial expression and the same 18 individuals showing a happy expression (see Fig 1). We computed continua of interpolated (morphed) faces using the Fantamorph software (Abrosoft Co.). Prototypical black and white pictures were taken from the Karolinska Directed Emotional Faces (KDEF) dataset [38]. These prototypical images were used as endpoints to generate a linear morph sequence with 15 steps, with intermediate images changing incrementally from unambiguously fearful to unambiguously happy, with emotionally ambiguous images in the middle. These 15 steps of morph were selected based on a pilot study (n = 19) to ensure that canonical expressions were recognized well above 75% and that ambiguous morph levels fell in the middle of the morph levels continuum. A grey mask surrounded each face. Luminance and contrast were equated for all faces. Each of the 18 actors * 15 morphs was repeated 3 times across the experiment to appear once in each emotional context, leading to a total of 810 trials. After each movie, we presented 45 semi-randomized trials of the decision-making task so that two consecutive morph degrees of the same actor were never presented in a row.

Each trial began with a fixation cross (500ms), followed by a morphed face (500ms) with the words “FEAR” and “HAPPY” presented below (order changed across subjects), all displayed on a grey background. Participants were forced to classify the faces as either fearful or happy, as accurately and as fast as possible. Inter-trial interval ranged between 500 and 1000ms. Faces were centered on the screen and subtended 8.6° of visual angle vertically and 6.7° horizontally.

### Analysis of responses

Within participants, the binary categorization of morph stimuli tends to increase monotonically from one response to the other across the morph continuum. The probability of answering “HAPPY” therefore takes the form of a standard sigmoid curve. We estimated the point of subjective equality (PSE) at which each participant was equally likely to classify faces as fearful or happy, by retrieving the morph degree corresponding to a probability of 50% of responding happy. We also estimated the slope of the curve at the PSE, which represents how sharp the distinction between fear and anger is. Data was fitted using a 4-parameter Weibull function. The lower and upper limits of the function were bounded to the interval [0, 1]. The shape parameter, which determines the steepness of the curve, was bounded to be greater than 1 to avoid non-sigmoidal shapes of the function. The slope and the PSE were computed using the drm package [39] implemented in R, specifying that the data was binomial (R Development Core Team, 2014). These parameters were estimated for each individual and for each emotional context, from the responses across the 15 morph degrees. Fits were obtained for all subjects except for one, for whom the fitting procedure could not converge in the fear and neutral contexts due to highly variable responses. This subject was thus excluded from all analyses.

Once PSEs and slopes for each emotional context and each subject were retrieved, we subtracted the value from each emotional context from that of the neutral context to obtain an estimate of PSE shift and slope change. Given our expression axis from fear to happy and our response axis in terms of probability of responding happy, a *rightward* (positive) shift in PSE indicated a bias toward classifying ambiguous faces as *fearful*, whereas a *leftward* (negative) shift indicated a higher propensity to classify ambiguous faces as *happy*. Note that because faces were the same across the 3 emotional contexts, PSE shifts cannot be attributed to differences in stimulus content or expression strength. For slope measurements, a positive (respectively negative) change indicated that sensitivity to discriminate between expressions increased (respectively decreased) between a given emotional context and the neutral context. PSE, slope, and reaction times were analyzed through repeated measure ANOVA and t-tests. Effect sizes were reported as Cohen’s d.

### Absorption ratings for movie stimuli

After the experiment, participants indicated how much they felt absorbed during each movie on a 10-point Likert scale (i.e. how much they felt emotionally immersed while watching each movie).

### Affect questionnaires

After the experiment, participants filled out questionnaires to rate their level of positive and negative mood in the last weeks (PANAS [40]), as well as current state anxiety (STAIS [41]), and depression [42]. In addition, trait anxiety (STAIT) was collected at least one day later. Because of technical reasons, questionnaire data was missing for one subject. Participants’ state anxiety scores ranged from 20 to 65 (mean±std 32.3±9.8) and trait anxiety scores ranged from 23 to 69 (40.9±9.3), which fall within the published norms for this age group [41]. Participants’ depression scores ranged from 0 to 16 (2.7±3.6), which is also below clinical levels for all subjects. As expected, scores for depression, state anxiety (STAIS), trait anxiety (STAIT), and negative mood (PANAS negative) were positively correlated between each other (all r>.63, all p < .001), and negatively correlated with scores for positive mood (PANAS positive) (all r<-.44 and all p < .002), except for the PANAS negative (r = -.25, p = .1). Influence of these variables on interpretation bias was examined using ANOVA and 1-tailed Spearman correlations were used in figures for illustrative purpose. In accordance with the literature on depression and anxiety [20–22], we expected that negative mood, depression and anxiety would be positively correlated with negative interpretation bias for facial expressions.

## Results

### Effects of transient emotions

**Fig 2:**
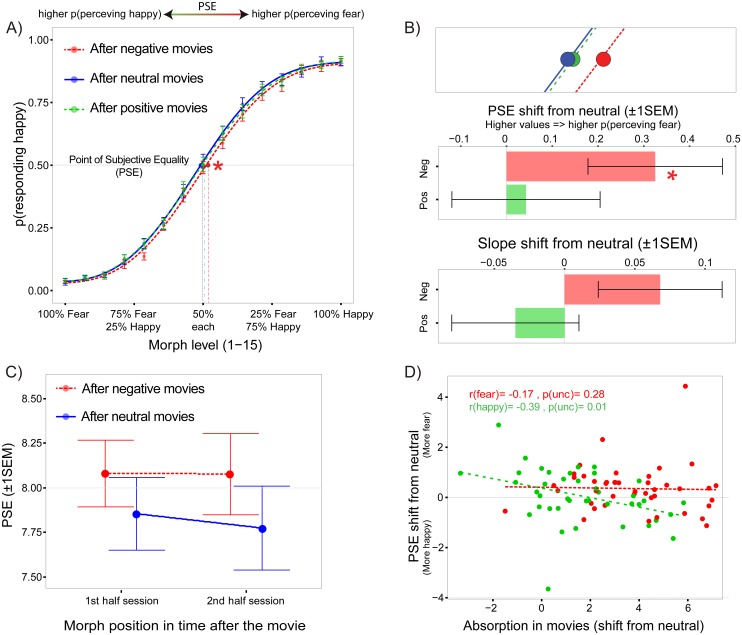
Interpretation bias following transient emotion induction. A) Sigmoid curves illustrating the probability of categorizing morphed faces as expressing happiness across morph levels and emotional contexts. Curve fitting resulted in two parameters for each subjects and each emotional context: the point of subjective equality (PSE), which characterizes the morph level at which the subject is at chance for discriminating happy vs. fearful expressions (also termed interpretation bias), and the slope of the curve at the PSE, which characterizes the sharpness of the decision boundary between happy and fearful faces. B) Top plot: zoom on the group average PSE for each emotional context. Middle plot: After viewing negative movies relative to neutral clips (red), participants increased their propensity to classify ambiguous expressions as fearful. No significant average shift (relative to neutral) was observed after watching positive movies (green). Bottom plot: There was an increase in slope after negative compared to neutral movies. C) Evolution of the bias over time in the negative context revealed that it persisted during the entire decision-making task (~2 minutes). Error represent ±1SEM. D) Correlations between absorption ratings and PSE shifts (for emotional compared to neutral movies) showed that for the positive context, more absorbing movies led to increased propensity to classify ambiguous faces as happy. Note that all statistical tests remained significant after removing two potential outliers with the most positive and the most negative PSE shifts.

We first tested for any significant shift in PSEs following emotional movies, relative to the neutral control condition. Planned t-tests on PSE shift across participants showed that ambiguous faces were more likely to be classified as fearful in the post-negative compared to the post-neutral movie context (t(42) = 2.21, p = .016, d = .19, 1-sided 1-sample t-test, Fig 2A and 2B). In contrast, positive context relative to neutral induced no PSE shift (p = .6).

Similar planned t-tests performed on slope changes showed no significant difference in either context relative to the neutral control condition (p’s > .13, 1-sample t-test). However, a pair-wise comparison between slope change in the negative versus positive contexts was significant (t(42) = 2.28, df(42), p = .02, paired t-test). Average change values revealed that the slope (i.e. sensitivity) of the discrimination performance was increased after exposure to negative compared to neutral movies (mean = .07±.28, d = .19), whereas this change was negligible after exposure to positive movies (mean = -.03±.29, d = .11) (see Fig 2B).

Next, we further characterized how the PSE bias observed in the negative context varied as the experiment unfolded. We divided each face perception blocks into first and second halves with respectively 22 and 23 stimuli each (Fig 2C). We re-computed PSE values for every subject for the negative and neutral contexts. We dropped the positive context from this analysis given the absence of results reported above. Because each fit was estimated on half the data, the fit procedure failed in two subjects who were excluded from this analysis. We entered the new PSE values in a repeated-measure ANOVA crossing the factors *time* and *emotion context*. The results confirmed a main effect of emotional context (F(1,40) = 5.67, p = .022). However, neither time (F(1,40) = .1, p = .74) nor the time-by-context interaction were significant (p = .72), indicating that the negative bias remained comparable during the entire face perception blocks and thus sustained over time.

In addition, we investigated whether absorption during movie watching influenced the PSE shift between emotional and neutral contexts. For negative movies, there was no correlation between the PSE shift magnitude and the difference in absorption between negative and neutral movies (r = -.17, df = 41, p = .28). However, in the positive context, the PSE shift was found to become significantly more positive as absorption in movies increased (r = -.39, df = 41, p = .01, Fig 2D). This suggests that subjects who felt more absorbed in positive movies favored more happy interpretation of ambiguous faces than subjects who were less absorbed. Note that positive and negative movies were rated as more absorbing than neutral movies (respectively t(42) = 5.1, p < .001 and t(42) = 11.8, p < .001), and negative movies more absorbing than positive movies (t(42) = 6.1, p < .001). Note also that the variance of absorption ratings for positive movies (sd = 1.87) tended to be higher than for negative movies (sd = 1.22) even though this difference was not significant (p = .77). This may suggest that interest in positive movies in our sample was more nuanced and variable than for negative movies.

### Effects of individual differences in mood, anxiety, and depression

To examine the effect of more persistent affective states and mood on facial expression perception, we ran four separate ANOVAs on the emotion-induced shift that tested respectively for modulations by positive mood, negative mood, depression, and anxiety, using the latter individual scores as a between-subject variable. These ANOVAs were run on PSE as the dependent variable and with emotional context as a within-subject factor.

**Fig 3:**
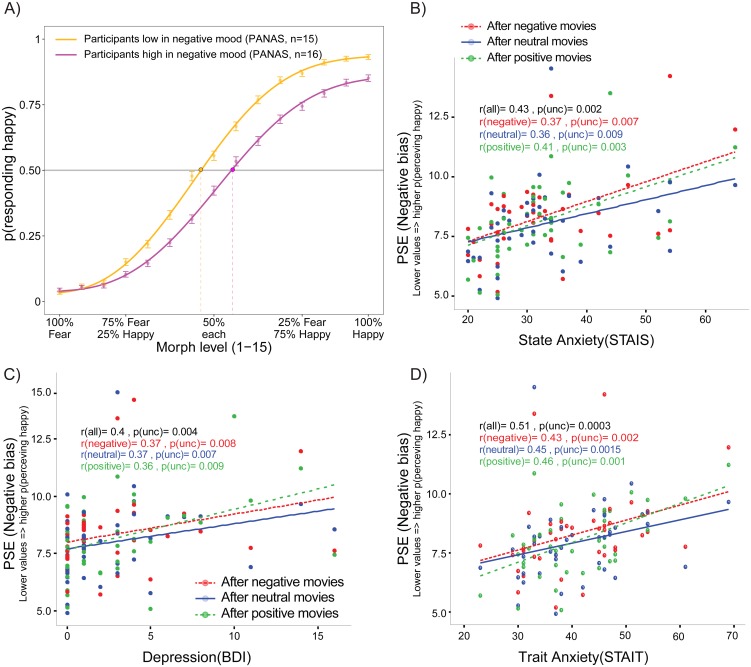
Interpretation bias are strongly influenced by persistent emotional states associated with individual trait differences. A) Subjects with a more negative mood showed a higher propensity to classify ambiguous faces as fearful than subjects with less negative mood (1^st^ and 3^rd^ tertiles of the PANAS negative scale). B-D) Correlations showing that the more individuals’ interpretation bias (i.e. PSE) was negative, the higher the state anxiety (B), depression (C), and trait anxiety (D).

These ANOVAs revealed no effect of positive mood (PANAS positive, p = .4). In contrast, there was a significant main effect of negative mood (PANAS negative, F(1,41) = 5.95, p = .02) but it did not interact with contextual factor (p = .92). Likewise, the third ANOVA with depression scores (Beck scale) indicated an effect of depression (F(1,41) = 4.2, p = .04) with no interaction with emotional context (p = .96). Finally, concerning anxiety scores (STAI-T and STAI-T), we found significant effects of both state anxiety (F(1,41) = 12.7, p < .001) and trait anxiety (F(1,41) = 8.7, p = .005), but again no interaction with emotional context (ps >.2). These data suggest that, in our subclinical population sample, the effect of emotion induction was independent of persistent affective background even though the latter did modulate emotion perception. Note that due to the high comorbidity between anxiety and depression, a multiple regression was not adapted to reliably disentangle the respective contribution of depression or anxiety, and it would be unlikely to reveal other results given the lack of significant effects in separate regression analyses. To illustrate the impact of negative PANAS on emotion perception, we divided participants in three equal groups (tertiles) according to the negative PANAS scores and we plotted the psychometric function of the groups with the lowest and highest negative mood scores in Fig 3A).

### Reaction times

Finally, we performed a repeated-measure ANOVA on reaction times (RT) during the decision-making task, with emotional context and morph levels as independent factors. Results indicated that RT evolved quadratically with morph levels (F(1,14) = 261, p < .001, quadratic contrast). This is reflecting more difficult judgments and longer latencies for more ambiguous faces close to the PSE (S1 Fig). The influence of emotional context was marginal (F(2,84) = 3.06, p = .052). There was no interaction between morph and context (p = .52) even though RT for rating emotional faces looked slower in the negative context (mean±SD: 962ms±283) than in the positive (890ms±204) or neutral contexts (921ms±237). Posthoc tests contrasting both emotional contexts confirmed that RT were slower in the positive than in the negative context (t(42) = 2.12, p = .04, d = .18). There was no significant difference between the neutral context and the negative (p = 08) and positive context (p = .4).

## Discussion

In this manuscript, we tested the hypothesis that several minutes of immersion into positive and/or negative emotional movies, may generate sustained affective states that outlast by ~2 minutes the exposure period and subsequently bias the interpretation of face expressions in a perceptual decision making task. In this sense, our work goes beyond recent priming studies by establishing a longer lasting impact of mood induction. We also investigated whether such effects are influenced by individual affect (depression and anxiety) traits. Finally, a key aspect of our study is the use of a psychometric approach to evaluate the processing of emotional faces allowing us to distinguish the source of the effect as a bias versus a change in sensitivity.

Our results demonstrate that transient emotional states modulate the interpretation of ambiguous emotional faces, with a stronger effect after watching negative movies than after positive movies. Induced negative states increased participants’ propensity to interpret ambiguous facial expressions in a more negative way relative to induced neutral or positive states. Interestingly, while positive movies did not modulate the interpretation of ambiguous expressions overall, participant’s propensity to rate faces as happy increased as their absorption during happy movies increased. In addition, we found that more persistent negative states related to individual traits, such as negative mood, anxiety, and depression, predicted the magnitude of the negative interpretation bias when judging ambiguous expressions, but did not influence the impact of the transient emotions induced by movies. This confirms that persistent negative affect leads to a tendency to over-interpret ambiguous information as negative [20–22]. The findings reported here therefore support the idea that transient and persistent emotional states ‘infuse’ cognitive states and influence evaluation in a state-congruent fashion [13]. They further extend previous work demonstrating that high trait absorption is related to enhanced emotional picture processing [33], by showing that state absorption can influence, at least for positive movies, the induction of a subsequent interpretation bias. They also show that such interpretation bias may remain present over several minutes, well beyond the short durations previously reported in the priming literature (where cue-target intervals of the order of hundreds of milliseconds are typically used).

Thus, these results add to previous evidence that information available during or shortly before perception of a target stimulus can bias its emotional evaluation [17,43,44]. According to affect priming models, emotional states can prime the encoding, retrieval, and selective use of information. They can also influence the evaluation of emotional stimuli and influence social judgments [13,15,16]. For instance, judgments related to quality-of-life, responsibility, future expectations, or political judgments are more optimistic after seeing a comedy, than after seeing a sad or a violent movie [45]. Certain priming procedures have used movies, music, or scripts to elicit emotional experiences. For instance, a previous experiment showed that the categorization of neutral, mildly happy, or mildly fearful faces was slightly influenced by the valence of a just preceding positive or negative IAPS picture [17]. Other experiments using the auditory modality showed that sad emotional states, induced by sad music, increase the propensity to rate ambiguous faces expressing various negative emotions as more negative [46,47], whereas joyful music induces the opposite effect [47,48]. Another experiment suggested that long lasting emotion effects could operate unconsciously by showing that subliminal happy face primes (compared to angry faces) may promote drinking of a pleasurable beverage during a subsequent unrelated task [49].

Our findings counter the idea that affective priming effects dissipate shortly after priming, as in paradigms where emotional primes (e.g. words) are presented for durations shorter than a few hundreds of milliseconds [10]. In such paradigms, it was argued that priming ceased to be detectable when the prime is presented beyond certain durations (typically 300ms). Moreover, these paradigms usually employ reaction time measurements, which may lack the sensitivity necessary to detect changes in affective processing. Note that we observed a global trend towards slower reaction times in the negative context relative to the positive context. A generic slowdown of reaction times in threatening contexts has often been interpreted as reflecting a freezing response or a suspension of ongoing task activity due to increased attentional vigilance towards the potential threat [50]. The reaction times changes were however noisy; an advantage of measuring shifts in interpretation bias, as we do here, is that they offer a very sensitive procedure to detect emotional priming effects. However, because this technique requires collecting decisions on several tens of trials, it can only detect biases that last for at least a few minutes. Our results establish that transient affective biases are not limited, nor stronger, during the immediate period following emotional events, and they may extend over a few minutes, at least when the emotional priming context has been presented for a sufficiently long time (e.g., in short movies here). Further work is needed to understand the relationship between the emotion induction duration, absorption during inducing events, and duration of the subsequently induced interpretative bias.

At the neural level, interpretation biases might result from an interaction between changes in the limbic system and interconnected regions in perceptual pathways and the prefrontal cortex. Notably, activity of the amygdala, a key center for emotion perception and fear processing, is modulated by the emotional information associated with or preceding the presentation of neutral faces [17]. Likewise, watching negative movies enhance subsequent amygdala reactivity to fearful faces, whereas positive movies attenuate it [5,35]. Similarly, when cued by a sentence with a negative context (e.g. he/she lost 500$), faces with a surprised expression produce greater activity in the ventral amygdala and the ventrolateral prefrontal cortex than when cued with positive sentences (e.g. he/she won 500$), while the latter produce stronger activity in the ventromedial prefrontal cortex [51]. Interestingly, Eryilmaz et al. (2011) observed that transient emotions can produce lasting changes in brain connectivity patterns, reflecting a reconfiguration of large-scale network states ([34], see also [36]). In particular, functional coupling between the amygdala and the ventromedial prefrontal cortex at rest is selectively reduced after watching negative emotional movies, similar to those used here [34]. Also, Pichon et al. (2012) showed that functional connectivity between the amygdala and the orbitofrontal cortex is reduced after priming with negative emotional words [19]. Hence, a modulation of amygdala functioning, together with its connectivity with other prefrontal and temporal regions, may provide a plausible neural substrate for explaining how emotional events influence subsequent affective processing and produce interpretation biases.

Similar to our findings, previous emotion induction procedures have shown that a negative context seems more efficient at priming a negative bias than a positive context is at inducing a positive bias [52–54]. In our study, the higher induction efficiency of negative over positive movies might be related to the fact that negative movies tend to depict universal representations (e.g., violence, death…), while positive movies and humor often rely on cultural norms which may vary between social groups and genders. This interpretation also accords with the fact that absorption ratings were generally lower and more variable for positive than negative movies. This suggests that tailoring the positive context to the targeted social groups or individuals, and carefully selecting or creating positive contexts that allow greater absorption, may be an important factor for regulating emotional responses towards more positive outcomes.

Persistent affective states such as mood, anxiety, and depression also impacted interpretation bias for facial expressions, even to a greater extent than transient emotional states (at least 5 times stronger in terms of effect size). Participants with high anxiety or subclinical depression displayed a higher propensity to interpret ambiguous faces as fearful. This is consistent with previous studies showing that depressed subjects display increased attention and memory toward negative stimuli [24,55]. Dysphoric individuals are more likely to classify morphed faces ranging from pure sadness to pure happiness as sad [23], and the magnitude of this bias correlates with their levels of depression, anxiety, and negative mood [56]. Also, anxious patients have a greater attentional vigilance to threatening stimuli and are more willing to interpret ambiguous situations as negative [57]. At the brain level, it is interesting to note that depressed patients show increased metabolic activity and altered grey matter volumes in amygdala-medial prefrontal networks [58], similar to those affected by transient negative priming effects as described above [36]. Hence, chronic dysfunction in this limbic-prefrontal network could lead to the maintenance of negative interpretation biases. Yet, direct evidence linking behaviour to brain function in this domain is still lacking.

However, despite these individual differences, we found no significant correlation between the persistent affective states and the impact of transient emotions on expression interpretation bias in our participants. Given that emotional blunting is frequent in depressed patients [59], whereas heighted emotional reactivity is commonly associated with anxiety [31], we expected a decreased or exaggerated emotion induction effect in subjects with higher depression or higher anxiety scores, respectively. This was not the case. It is worth noting that none of our subjects were highly depressed or clinically anxious. Sampling over a wider spectrum of depression or anxiety levels might be necessary for testing this hypothesis.

In conclusion, the present work shows that both transient and persistent affective states influence the way we perceive emotionally ambiguous information. The decision-making task we introduce here shows enough sensitivity to repeatedly detect shifts in emotion expression as a function of different emotional contexts. In addition, given that this perceptual measure correlates well with anxiety and depression scores, it could be used to provide a quantitative and implicit evaluation for the outcomes of an intervention. For example, such a test could be used to assess the transfer of learning after cognitive therapy, which aims to train patients to favor positive interpretations of situations over negative ones [60]. An interesting extension of the present work would be to better characterize how the duration of priming events and the absorption experienced during them may determine the strength, persistence, and volatility of the subsequently induced biases. It would also be interesting to assess whether repeated exposures to specific priming contexts (such as emotionally charged videogames) could lead to more persistent interpretation biases.

## Supporting information

S1 Fig Mean reaction times across morph levels revealed longer latencies for more ambiguous expressions around the PSE. Reaction times in the negative context were slower than in the positive context (negative: red dotted line, neutral: blue plain line, positive: green dotted line).
(TIF)
