# Supplementary figures and images for: Transient emotional events and individual affective traits affect emotion recognition in a perceptual decision-making task
Source: PLoS One. 2017 Feb 2;12(2):e0171375. doi: 10.1371/journal.pone.0171375 (PMC5289590; doi:10.1371/journal.pone.0171375)

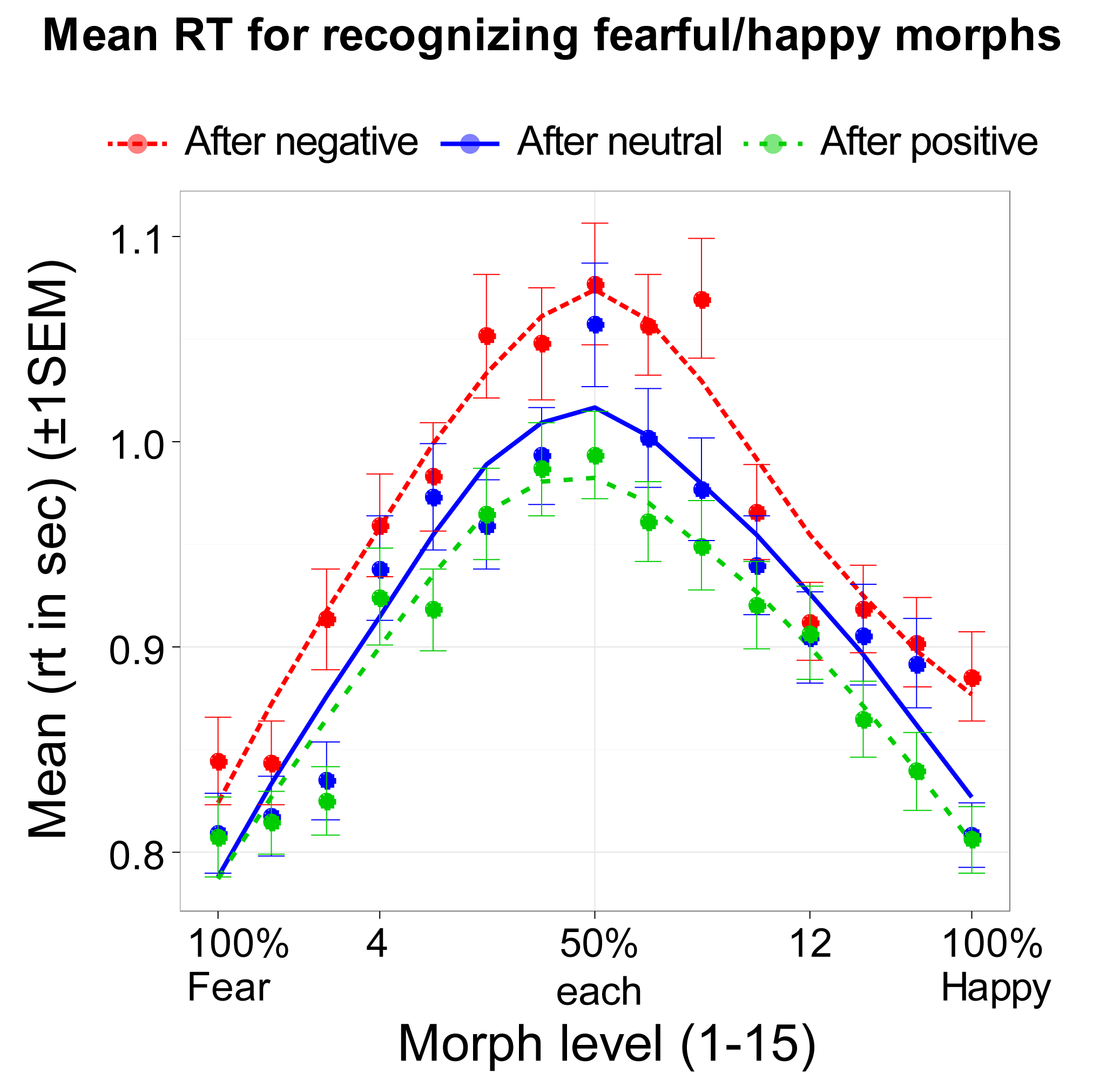

Supplement: S1 Fig — Reaction times in the negative context were slower than in the positive context (negative: red dotted line, neutral: blue plain line, positive: green dotted line). (TIF) [file pone.0171375.s001.tif]
